# The effect of optic neuritis treatment trial (ONTT) combined corticosteroid regimen on pattern reversal visual evoked potentials: a prospective follow-up study

**DOI:** 10.1186/s12886-021-02086-z

**Published:** 2021-09-06

**Authors:** Padmini Dahanayake, Tharaka L. Dassanayake, Manoji Pathirage, Saman Senanayake, Mike Sedgwick, Vajira S. Weerasinghe

**Affiliations:** 1 Department of Physiology, Faculty of Medicine, University of Peradeniya, Peradeniya, 20400 Sri Lanka; 2 Teaching Hospital, Peradeniya, 20400 Sri Lanka; 3 School of Psychology, The University of Newcastle, Callaghan, NSW 2308 Australia; 4 Department of Medicine, Faculty of Medicine, University of Peradeniya, Peradeniya, 20400 Sri Lanka; 5 Eye Unit, National Hospital, Kandy, 20000 Sri Lanka

**Keywords:** Visual evoked potentials, P100, Prospective study, Optic neuritis, ONTT combined regimen, Corticosteroids, Visual conduction, Remyelination

## Abstract

**Background:**

The combined corticosteroid regimen of the original Optic Neuritis Treatment Trial (ONTT) is used in many centers to treat optic neuritis. Though pattern reversal visual evoked potentials (PRVEPs) are a sensitive, standard measure of visual conduction in optic neuritis, no studies *hitherto* have investigated the effect of combined ONTT regimen on PRVEPs. We aimed to determine the effect of combined corticosteroid regimen of the ONTT on changes of PRVEPs in patients with first-episode optic neuritis over 3 months post-treatment.

**Methods:**

This is a prospective, observational study in which 44 patients with optic neuritis were seen pre-treatment (baseline) and follow-up, at 1 month (FU1) and 3 months (FU2). Twenty-nine patients were treated with ONTT combined regimen (ONTT+ Group) while 15 were conservatively managed without corticosteroids (ONTT- Group). The median latency and amplitude values of the P100 PRVEP component and the visual acuity (i.e. LogMAR values) at pre-treatment, FU1 and FU2 were compared in the two groups using Friedman’s rank test and Wilcoxon Signed Ranks test.

**Results:**

Median P100 latency improved significantly (to the normal range) as early as by 1 month after the commencement of treatment in the ONTT+ Group, and then remained significantly lower than the baseline over next 2 months. In the ONTT- Group, the median P100 latency improved more slowly over the two follow up assessments and reached the normal range by 3 months. Median visual acuity values also improved significantly at 1 and 3 months after the commencement of treatment in the ONTT+ Group but not in the ONTT- Group.

**Conclusion:**

ONTT combined corticosteroid regimen improves conduction in the visual pathways of patients with first-episode optic neuritis earlier than does conservative management. We provide electrodiagnostic evidence that combined ONTT regimen–compared with conservative management–results in early remission of visual conduction abnormalities in first-episode optic neuritis.

## Background

Optic neuritis is characterized by an inflammation and demyelination of the optic nerve [1], and is traditionally treated with corticosteroids. Pattern-reversal visual evoked potentials (PRVEPs) are a sensitive measure to assess conduction delay in the optic nerve pathway caused by demyelination [2]. The Optic Neuritis Treatment Trial (ONTT) conducted in a large sample of patients over a 15-year follow up starting from 1988, found a combination of intravenous and oral corticosteroids produced rapid recovery, a better short-term functional outcome and reduced recurrence of optic neuritis in multiple sclerosis [1]. Since then, combined corticosteroid regimen has become the standard treatment for optic neuritis. The outcome measures of the study were visual acuity, contrast sensitivity, colour vision and visual field testing. Although these are clinically important functional outcome measures, objective assessment of the conduction along the optic pathways using visual evoked potentials (VEPs) would add to the clinicians’ understanding of the recovery of their patients [3, 4].

Given that VEPs were not used as an outcome measure in the original ONTT study, or to best of our knowledge, in any subsequent combined corticosteroid trials, we used pattern reversal VEPs (PRVEPs) to assess the short-term recovery of the visual conduction pathways in patients with optic neuritis who underwent the combined ONTT regimen. Specifically, we assessed the PRVEPs of two cohorts of patients with newly-diagnosed, first-episode optic neuritis who underwent 1) combined ONTT regimen or 2) conservative management with no corticosteroids at three time points: pre-treatment (baseline); and 1 month and 3 months following commencement of clinical management.

Previous studies have employed VEPs in assessment of recovery of optic neuritis following either oral or intravenous corticosteroid treatment [5–10], but none have assessed the effect of combined ONTT regimen on the recovery of VEPs. We also compared the baseline VEP measures of the whole group of patients with a reference group of healthy individuals.

## Methods

### Study setting and design

This was a prospective, observational study carried out from February 2017 to October 2019 at the Ophthalmology Centre of the National Hospital Kandy and the Clinical Neurophysiology Department of the Teaching Hospital Peradeniya, two tertiary care referral centres of Sri Lanka. The study design and protocols complied with the code of ethics of the World Medical Association Declaration of Helsinki [11]. Ethical clearance for the study was obtained from the Ethical Review Committee, Faculty of Medicine, University of Peradeniya, Sri Lanka. The procedure was explained, and informed written consent was obtained from the participants recruited in the study.

### Characteristics of participants

Forty-four patients ≥18 years of age with the diagnosis of the first-episode optic neuritis within 3 weeks after onset of ocular symptoms were recruited. Participants were excluded if they had received corticosteroids in the previous 30 days, had other neurological illnesses, or other medical conditions that might affect visual outcomes such as, but not limited to, diabetic retinopathy, glaucoma, or cataracts. One of the co-authors (SS), who is a specialist ophthalmologist clinically examined and conducted fundoscopic examination of the potential participants. Patients were diagnosed based on sudden onset of visual impairment, positive relative afferent pupillary defect (RAPD), fundoscopic changes, and PRVEP changes (which we used as the baseline VEP measurements). None of the recruited participants had fundoscopic features of other eye pathologies that might confound our results.

To compare the baseline VEP measures of the above group with optic neuritis, we also recruited a reference group of 44 healthy participants of age ≥ 18 years with normal or corrected-to-normal vision in neuro-ophthalmologic examination. They had no concomitant or previous eye diseases and no evidence of neurological diseases.

### Procedure

All the participants (test and healthy reference groups) had a baseline neuro-ophthalmologic assessment which included measurements of visual acuity, visual field, colour vision, pupillary reflexes, fundoscopy and ocular motor examination.

The best corrected visual acuity was examined using a Snellen chart and converted into a logarithm of the Minimal Angle of Resolution (LogMAR) units for statistical analysis. We tested all participants with pattern electroretinography (PERG) to exclude possible macular pathology (as indexed by delayed P50 PERG component latency) that might delay VEPs despite the post-retinal pathways being intact, and thus 1) to exclude individuals with macular pathologies from the healthy reference group and 2) to prevent patients with isolated macular pathologies being recruited erroneously into the optic neuritis group [12]. We have reported the PERG assessment techniques in detail previously [13].

All participants underwent PRVEPs assessments at the Clinical Neurophysiology Department of the Teaching Hospital Peradeniya. A Natus EMG/NCV/EP machine (Natus Neurology Inc. USA) was used to produce visual stimuli, and to record and average the PRVEP waveforms. The recording technique conformed to the International Society for Clinical Electrophysiology of Vision (ISCEV) guidelines [14]. PRVEPs were recorded in response to 2 Hz pattern-reversal checkerboard stimuli presented on a cathode ray tube monitor with a black-and-white alternating checkerboard pattern. The field size subtended and visual angle 15° at the shortest diagonal. The contrast between black and white squares was ≥80% as defined by Michelson contrast. Silver/silver chloride electrodes were used to record VEPs. An active electrode was fixed at the Oz position, reference electrode at Fz position and ground electrode at Cz position according to the 10/20 electrode placement standards. The electrode impedances were maintained below 5 kΩ. Mean photopic luminance was 50 cdm^− 2^. The mean luminance of the stimulus screen was constant during checkerboard reversals and varied less than 30% between the center and periphery of the visual field. Each eye was tested separately. A band-pass filter with low- and high-cutoff frequencies of 1 Hz and 100 Hz was applied. One hundred sweeps were averaged online, and two averaged waveforms were recorded to check for reproducibility. The peak amplitudes and latencies of the N75, P100 and N145 components were measured.

### Treatment and follow up

A baseline (pre-treatment) PRVEP measurement was done in all patients with optic neuritis and the reference group of healthy individuals.

**Fig. 1:**
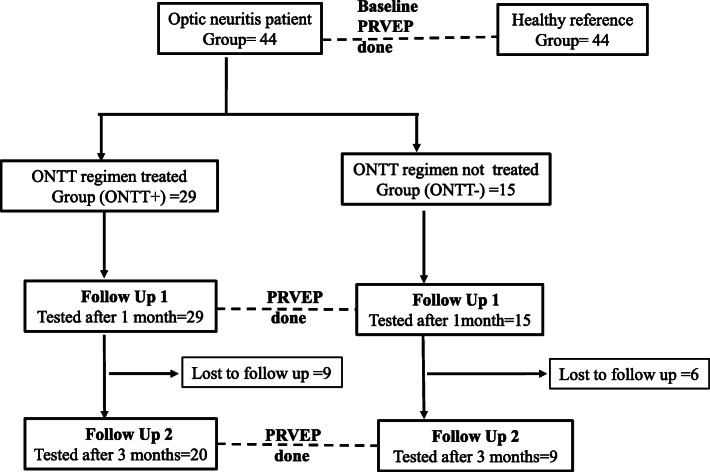
Participants recruitment and follow up

The decision to treat with ONTT regimen or to manage conservatively was taken by the treating ophthalmologists on an individualized basis [15, 16]. Of the 44 patients, 29 were treated with the full course of ONTT regimen (ONTT+ Group) which was 3 doses of IV methylprednisolone (1 g/day) followed by 11 days of oral prednisolone (1 mg/kg/day) with a 3-day short oral tapering course [1, 16, 17]. The remaining 15 patients were not treated with ONTT combined regimen, and were managed conservatively (ONTT- Group) [18]. Each cohort was followed up with two subsequent assessments using PRVEP: after 1 month (FU1) and then after 3 months (FU2) after commencement of treatment (Fig. 1).

### Data analysis

PRVEP latencies and amplitudes were the electrophysiological outcome measures. The primary PRVEP measure was the P100 latency of PRVEP because P100 latencies are a reliable measure of the functional integrity of the visual pathway. P100 latency was also found to correlate with visual acuity and recovery of visual fields in patients with optic neuritis [5, 19]. In addition, visual acuity reported in LogMAR units were considered the main clinical outcome measure. These outcome measures showed skewed distributions; and therefore, are reported as medians and interquartile ranges (IQR), and were analysed using non-parametric tests. In four VEP assessments (of the patient group) the P100 VEP component was absent. In those cases, a dummy value of 999 ms was assigned for these entries to retain those cases in the samples and thus avert the group’s statistics biasing towards better values. Given the number of such cases were a small fraction and comparisons are nonparametric, this approach did not affect the median and IQRs [5]. In addition to the primary outcome measures, we also report the latencies and amplitudes of N75 and N145 components.

Baseline PRVEP comparisons between the whole group of patients and the healthy reference group was conducted using Mann-Whitney U test. The outcome measures among the baseline and follow up assessments (FU1 and FU2) were compared within each treatment group (i.e. ONTT+ and ONTT-) separately, with Friedman’s ranks test, the nonparametric equivalent of repeated measures one-way analysis of variance. If significant difference was observed in Friedman’s ranks test in a group, three *post-hoc* paired comparisons were done using the Wilcoxon Signed Ranks test to determine the difference in baseline vs. FU1, FU1 vs. FU2 and baseline vs. FU2 assessments. The above *post-hoc* comparisons were done with a modified Bonferroni correction procedure–the stepwise Hochberg approach–to test statistical significance. The procedure ranks the *p*-values (three p-values in this study) and tests the first (lowest p-value) at 0.05/3. If that is significant, the procedure then tests the next one at 0.05/2; and if that becomes significant, the procedure tests the next one at 0.05/1. If one of the *p*-values is not significant, all those below that on the ranking are not significant [20, 21]. IBM SPSS Statistics for Windows, version 22.0 was used to analyse the data.

## Results

### Demographic and clinical characteristics

Figure 1 shows the numbers of participants initially recruited and retained at each stage of the follow up. There were 44 patients with optic neuritis (24 females; median age: 47.5 (IQR: 37.2–53; range: 18–70) years. All patients had a clinical history of sudden onset visual disturbances, most frequently blurred vision. These patients underwent baseline visual electrophysiological assessment after a median duration of 10 (IQR: 7–20; range 2–21) days following onset of symptoms. Twenty-seven patients complained of unilateral visual impairment while 17 complained of bilateral impairment. The worst clinically affected eye in each patient was taken as the *affected eye*. The clinical and electrophysiological findings reported and analysed in this paper are of the affected eye of each patient.

Ten patients had normal fundoscopy and 34 showed optic disc edema of the affected eye. Nineteen had undergone magnetic resonance imaging (MRI) and four showed MRI features of multiple sclerosis. Twenty-eight (63.6%) had ocular pain in the affected eyes at the onset of the clinical symptoms, but none had pain at the time of the visual electrophysiological examination. RAPD was positive in 36 (81.8%) patients. Median N75, P100 and N145 latencies in PRVEP were significantly prolonged in the affected eye of the patient group compared with the healthy reference group; and all 44 patients had P100 latency above 110 ms, our upper limit of normal, signifying demyelinating optic neuritis (Table 1).

**Table 1:** Comparison of baseline PRVEP measures between optic neuritis patients and healthy individuals

PRVEP measure	Median (IQR)		*P* value (Mann-Whitney U test)
	Optic neuritis patient Group (*n* = 44)	Healthy reference Group (*n* = 44)	
N75 Latency	95.75 (79.5–106.5)	74.9 (71.9–79.2)	**< 0.001**
P100 Latency	126.5 (120.0–139.5)	100.1 (97.6–106.4)	**< 0.001**
N145 Latency	159.5 (149.9–175)	139.3 (135–142.7)	**< 0.001**
N75 Amplitude	1.0 (0.5–1.0)	0.9 (0.5–1.4)	0.983
P100 Amplitude	5.0 (3–8)	3.5 (2.5–6.9)	0.099
N145 Amplitude	4.6 (2.5–6.6)	4.0 (2.8–5.9)	0.900

There were 29 patients (16 females) in the ONTT+ Group. Their median age was 49 (IQR: 37–53; range: 18–70) years. All 29 attended the FU1 assessment while only 20 (69%) patients were followed up at the FU2. The ONTT- Group consisted of 15 patients (8 females). Their median age was 47 (IQR: 37–57; range: 22–62) years. All 15 patients were assessed at the FU1, but only nine patients (60%) attended the FU2. There were no significant differences in the baseline characteristics between the ONTT+ and ONTT- Groups (Table 2).

**Table 2:** Baseline characteristics of ONTT+ vs ONTT- Groups

Baseline characteristics	ONTT+ Group	ONTT- Group	*P* value
Age (Median (IQR) in years^a^	49 (37–53)	47 (37–57)	0.673
Proportion (%) of females^b^	16/29 (55.2%)	8/15 (53.3%)	1.000
Visual acuity (Median (IQR)^a^	0.6 (0.5–0.6)	1.0 (0.5–1)	0.064
Proportion with eye pain (%)^b^	20/29 (69%)	8/15 (53.3%)	0.340
Proportion with disc edema (%)^b^	22/29 (75.9%)	12/15 (80%)	1.000
Median (IQR) P100 latency (ms)^a^	127 (120.5–143.3)	126 (118.3–136)	0.683

^a^Mann-Whitney U test, ^b^Fisher’s Exact test

### PRVEP measures in ONTT+ Group vs. ONTT- Group

**Fig. 2:**
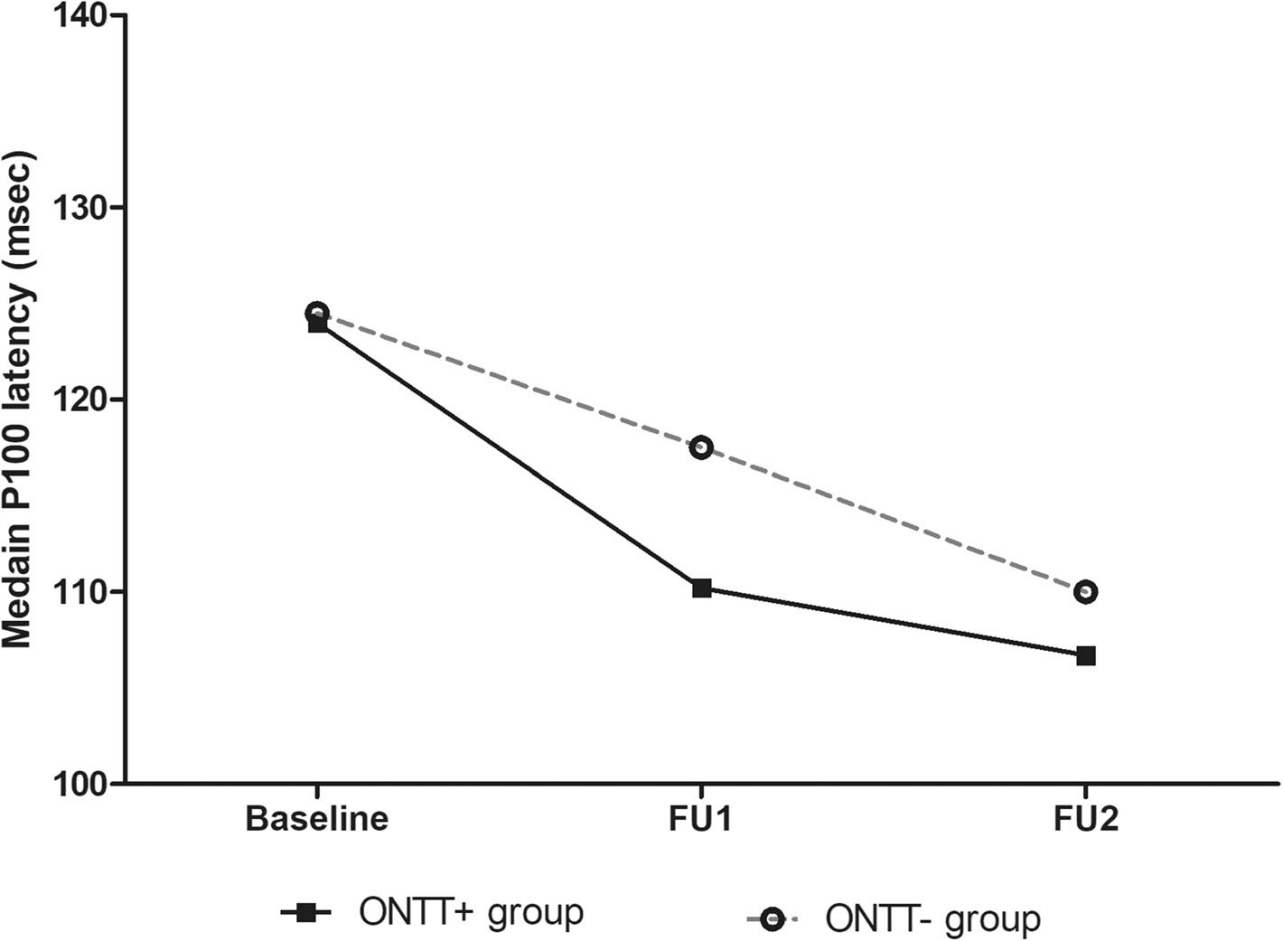
The median P100 latencies in the ONTT+ and ONTT- Groups at baseline and follow-up assessments

In the ONTT+ Group, there was a significant improvement in the median P100 latency values of affected eyes both at 1 month and 3 months after the commencement of treatment. Specifically, the median P100 improved to the normal range as early as 1 month after the commencement of treatment, and the remission lasted until the second follow up assessment at 3 months (Table 3 and Fig. 2).

**Table 3:** Comparison of PRVEPs in affected eyes between baseline, FU1and FU2 assessments in ONTT+ Group

PRVEP measure	Median (IQR)			Overall *p* value (Friedman’s rank test)	*P* value (Wilcoxon’s Signed Ranks test)		
	Baseline (*n* = 29)	FU1 (*n* = 29)	FU2 (*n* = 20)		Baseline vs. FU1 (*n* = 29)	FU1 vs FU2 (*n* = 20)	Baseline vs. FU2 (*n* = 20)
N75L	97.25 (79.8–106.4)	78.4 (69.6–85.8)	74.1 (60.9–86.6)	**0.021**	**0.008**	0.472	**0.003**
P100L	124.4 (121–142.7)	110.9 (98.2–123.2)	106.7 (94.6–119.8)	**0.001**	**< 0.001**	0.171	**< 0.001**
N145L	160.1 (148.5–178.6)	151.0 (138.1–162.9)	151.2 (132.7–158.2)	**0.016**	**0.012**	0.732	**0.008**
N75A	0.7 (0.3–1)	1.0 (0.25–1)	0.5 (0.2–1.0)	0.476	0.303	0.740	0.434
P100A	4.7 (2.8–6.7)	4.6 (2.1–7)	3.75 (2.3–5.5)	0.534	0.746	0.177	0.456
N145A	3.5 (2.5–6.5)	3.4 (1.7–4.4)	4.25 (2.9–5.9)	0.157	0.50	0.204	0.948

*L* Latency, *A* Amplitude, *IQR* Inter Quartile Range

In the ONTT- Group, there was a significant improvement in median P100 latency values in both the FU1 and FU2 assessments compared to the baseline assessment (Table 4). But the reduction over the 3 months post-baseline was more linear, with partial improvement after 1 month and a further reduction at 3 months (Fig. 2).

**Table 4:** Comparison of PRVEPs in affected eyes between baseline, FU1and FU2 assessments in ONTT- Group

PRVEP measure	Median (IQR)			Overall *p* value (Friedman’s ranks test)	Paired comparisons *p* value (Wilcoxon’s Signed Ranks test)		
	Baseline (*n* = 15)	FU1 (*n* = 15)	FU2 (*n* = 9)		Baseline vs. FU1 (*n* = 15)	FU1 vs FU2 (*n* = 9)	Baseline vs. FU2 (*n* = 9)
N75L	95.0 (73.4–108.4)	81.5 (72.9–86.6)	76.5 (61.5–90.9)	0.272	0.026	0.735	0.066
P100L	124.5 (117.6–135.2)	117.5 (108.2–122.5)	110.0 (103–125)	**0.007**	**0.008**	0.407	**0.007**
N145L	160.8 (154.4–172.1)	156.0 (144.9–177.0)	149 (142–167.1)	0.293	0.055	0.155	0.038
N75A	1 (0.5–1.15)	1.0 (0.5–2)	1.0 (0.2–1.0)	0.391	0.556	0.128	0.553
P100A	6.0 (3.2–9.7)	6.5 (3–8.5)	6.0 (3.7–8.7)	0.539	0.706	1.000	0.678
N145A	6.5 (3.9–9.0)	6.0 (3.5–11.9)	6.5 (3–8.3)	0.913	0.451	0.612	0.678

*L* Latency, *A* Amplitude, *IQR* Inter Quartile Range

There was a significant improvement in LogMAR values of visual acuity at FU1 assessment and further improvement was noted by FU2 in the ONTT+ Group. In the ONTT- Group, there was a trend of improvement in median LogMAR values of visual acuity over the 3 months duration, but the differences were not statistically significant which might be due to the limited number of subjects (Table 5).

**Table 5:** Comparison of visual acuity across baseline, FU1 and FU2 assessments in ONTT+ and ONTT- Groups

Study Group	Visual acuity LogMAR median (IQR)			Overall *p* value (Friedman’s rank test)	*P* value (Wilcoxon’s Signed Ranks test)		
	Baseline	FU1	FU2		Baseline vs. FU1	FU1 vs FU2	Baseline vs. FU2
ONTT+	+ 0.6 (0.5–0.6)	+ 0.3 (0.0–0.6)	+ 0.2 (0.0–0.45)	**< 0.001**	**0.001 (*n*** **= 29)**	0.076 (*n* = 20)	**0.001 (*n*** **= 20)**
ONTT-	+ 1.0 (0.5–1.5)	+ 0.8 (0.1–1.65)	+ 0.2 (0.05–1.4)	0.013	0.023 (*n* = 15)	0.068 (*n* = 9)	0.027 (*n* = 9)

However, in a subsidiary analysis, we did not observe a significant correlation between improvement of P100 latency and LogMAR visual acuity with time in any of the two groups (Table 6). Furthermore, we determined the proportions of patients who had improvement in P100 latency values to normal range which was considered as < 110 ms. In the ONTT+ Group, 11/29 (38%) and 12/20 (60%) patients showed improvement in P100 latency values into normal range at FU1 and FU2 respectively. In the ONTT- Group, 6/15 (40%) and 5/9 (55.5%) improved to normal range in P100 latency values at FU1 and FU2 respectively. However, a statistical comparison of two groups was not done since the number of each group was low.

**Table 6:** Correlation between improvement in VEP P100 latency and LogMAR visual acuity at follow up assessments

Comparison	Spearman correlation coefficient (*p* value)	
	ONTT+ Group	ONTT- Group
Baseline to follow-up 1	0.11 (0.56)	0.46 (0.09)
Baseline to follow up 2	−0.03 (0.91)	0.29 (0.44)

## Discussion

In the present study, we observed a significant improvement in median P100 latency values in PRVEP both in the group treated with the ONTT combined regimen and the conservatively managed group. However, the time course of improvement was different in the two groups. In the ONTT combined regimen treated group, the median P100 latency value improved to the normal range 1 month after commencement of treatment. In this group the P100 latencies remained significantly lower than the baseline over next 2 months, but with no further latency reduction over and above that was observed at 1 month. The pattern of P100 latency changes in the conservatively managed group was different: The reduction over the 3 months post-baseline was more linear, with partial improvement after 1 month and a further improvement at 3 months. Although direct intergroup comparisons were not done due to limited sample sizes, the overall improvement tends to be greater in the group treated with ONTT combined regimen (Fig. 2). In combination these observations indicate ONTT regimen, compared to conservative management, achieves early remission in visual conduction abnormalities in optic neuritis.

Our findings are consistent with those of the original ONTT study and other prospective studies that investigated the effect of combined ONTT regimen on visual acuity, colour vision, visual fields and contrast sensitivity in patients with optic neuritis [1, 22–24]. The follow up data from the original ONTT study shows that the combined ONTT regimen elicits a significant improvement in visual acuity, contrast sensitivity and visual fields sooner than the placebo group and the group treated with oral prednisolone [1, 25]. In that study, combined ONTT regimen elicited a significant improvement in visual acuity, contrast sensitivity and visual fields by 1 month and then the rate of improvement gradually declined. By around 3 months, about 50–70% of the patients had normal visual outcome measures. Similar to the present findings, the groups that received placebo and oral prednisolone showed a more linear recovery. Our findings provide additional electrophysiological evidence supporting more rapid improvement of visual conduction in optic neuritis in the group treated with combined corticosteroids.

Although the effect of combined ONTT regimen on PRVEP has not been assessed before, a few follow up studies have assessed the effectiveness of either oral or intravenous corticosteroids on PRVEP in patients with optic neuritis [5–10] (Table 7). Of those, one randomized controlled trial has shown that oral corticosteroids improve P100 PRVEP latencies by 1 month [5], whereas another randomized controlled trial has shown that oral corticosteroids can be used as an alternative to IV corticosteroids to treat acute optic neuritis [7]. Another randomized controlled trial has shown that no PRVEP improvement occurs with IV corticosteroids even by 3 months [6]. Similarly, a case series suggests that IV corticosteroids improve P100 PRVEP at 3 months [9], while another series has shown that IV corticosteroids are not associated with recovery of PRVEP by 1 month [8].

**Table 7:** Summary of studies that assessed PRVEP changes following corticosteroid treatment in optic neuritis

Study & Country	Type of study	Number of participants	Duration of symptoms	Route of administration, dose and duration	Follow up	Improvement in visual acuity	Improvement in PRVEP P100 latency
Smith et al., 1986 [8] Denmark	Case series	10	Within 24 h of onset	IV Methylprednisolone 1 g daily for 3 days	1 week and 1 month	Not quantified.	No significant improvement; mean (SE) Before: 132 (3.3) 1 week: 127 (5.8) 1 month:134 (3.2)
Trauzettel-Klosinski et al., 1995 [5] Germany	Randomized controlled trial	Test group: 15 Placebo: 33	3 weeks since onset	Test group: oral Methylprednisolone 100 mg daily for 3 days followed by tapered dose reduction in every 3 days up to 24 days. Placebo: oral thiamine 100 mg daily for 24 days	1 week, 1, 3 and 12 months after onset	1 week: Improved in both groups. Slightly in favor of oral group without statistical significance 3 and 12 months: no significant difference in improvement in both groups 12 months: normalized in most patients in both groups	*Between group analysis*: 1 month: Significant improvement in the test group. 3 and 12 months: no significant difference in improvement between two groups. *within-subject analysis* *Test group* 1 month: borderline significant improvement 3 and 12 months: no significant difference in improvement *Placebo group* no significant difference of improvement over the time
Kapoor et al., 1998 [6] United Kingdom	Randomized controlled trial	Test group: 33 Placebo: 31	Within 30 days of onset	IV Methylprednisolone 1 g daily for 3 days Placebo: Normal saline for 3 days	2 weeks, 3 months and 6 months	Not quantified	IV corticosteroids have no effect on VEP latency improvement at 2 weeks, 3 and 6 months
Sethi et al., 2006 [9] India	Case series	33 eyes	Within 2–15 days	IV dexamethasone 100 mg for 3 days	3 months	3 months: VA ≥ 20/20 achieved in 16 (57.14%) eyes	Statistically significant improvement was seen at 3 months with IV corticosteroids
Morrow et al., 2018 [7] Canada	Randomized controlled trial	IV group: 23 Oral group: 22	Within 14 days of onset	IV Methylprednisolone 1000 mg daily for 3 days Oral prednisolone 1250 mg daily for 3 days	1 and 6 months	IV Group: median Baseline: 20/100 1 month:20/20 6 months:20/20 Oral Group: median Baseline: 20/160 1 month:20/36 6 months:20/32 recovery not significantly different between the groups	IV Group: Mean (SD) *Baseline:* 181.9 (53.6) 1 month: 140.1 (28.5) 6 months: 119.0 (16.5) *Oral Group*: Mean (SD) Baseline: 200.5 (67.2) 1 month: 145.4 (41.5) 6 months: 133.8 (31.5) no significant difference in improvement between the groups. Thus, oral corticosteroids can be used as an alternative to IV corticosteroids to treat acute optic neuritis

PRVEP latency prolongation is a measure of the degree of demyelination of the visual conduction pathways: recovery of latencies therefore signifies remyelination. Improvement of the PRVEP latencies that we observed in patients with optic neuritis is due to the remyelination process, and restoration of ionic conductance along the optic nerve pathways [26, 27]. Furthermore, early treatment with steroids in optic neuritis was found to minimize axonal loss as indexed by retinal nerve fiber layer thickness assessments with optical coherence tomography [28]. Fluorescence microscopy on experimental animals has shown that corticosteroids can suppress optic neuritis and prevent retinal ganglion cell loss, if corticosteroid treatment is initiated in the early phase of the disease [29].

Our study, however, is limited in assessing the retinal morphology as we could not measure the retinal layer thicknesses with optical coherence tomography in the present study. Another limitation of our study is that it was underpowered to compare the proportions of patients who had complete recovery of VEPs between the two treatment arms, owing to small number of subjects.

## Conclusions

To the best of our knowledge, present study is the first to evaluate the efficacy of ONTT combined regimen on the recovery of optic neuritis compared with conservative management as assessed by PRVEP. We conclude that management of patients either conservatively or with combined ONTT regimen improves visual conduction significantly at 3 months, though the ONTT combined regimen accelerated the recovery, showing a clinically significant improvement of visual conduction as early as 1 month.

## Data Availability

The data sets used and/or analysed during the current study are available from the corresponding author on request.

## Abbreviations

EMG: Electromyography
IQR: Inter quartile range
ISCEV: International society for clinical electrophysiology of vision
LogMAR: Logarithm of the minimal angle of resolution
MRI: Magnetic resonance imaging
PERG: Pattern electroretinography
PRVEP: Pattern reversal visual evoked potentials
